# On the origin of European sheep as revealed by the diversity of the Balkan breeds and by optimizing population-genetic analysis tools

**DOI:** 10.1186/s12711-020-00545-7

**Published:** 2020-05-14

**Authors:** Elena Ciani, Salvatore Mastrangelo, Anne Da Silva, Fabio Marroni, Maja Ferenčaković, Paolo Ajmone-Marsan, Hayley Baird, Mario Barbato, Licia Colli, Chiara Delvento, Toni Dovenski, Gregor Gorjanc, Stephen J. G. Hall, Anila Hoda, Meng-Hua Li, Božidarka Marković, John McEwan, Mohammad H. Moradi, Otsanda Ruiz-Larrañaga, Dragana Ružić-Muslić, Dragica Šalamon, Mojca Simčič, Ondrej Stepanek, Econogene Consortium, Sheephapmap Consortium, Ino Curik, Vlatka Cubric-Curik, Johannes A. Lenstra

**Affiliations:** 1 Dipartamento Bioscienze, Biotecnologie, Biofarmaceutica, Universita. degli Studi di Bari “Aldo Moro”, Bari, Italy; 2 Dipartimento Scienze Agrarie, Alimentari e Forestali, Universita Studi di Palermo, Palermo, Italy; 3 Université de Limoges, INRAE, Pereine EA7500, USC1061 Gamaa, 87000 Limoges, France; 4 Dipartamento Scienze Agroalimentari, Ambientali e Animali, Universita Udine, Udine, Italy; 5 Department of Animal Science, University of Zagreb, Zagreb, Croatia; 6 Dipartimento di Scienze Animali, della Nutrizione e degli Alimenti, Universita Cattolica del S. Cuore di Piacenza, Piacenza, Italy; 7 AgResearch, Invermay Agricultural Centre, Mosgiel, New Zealand; 8 Department of Reproduction and Biomedicine, Faculty of Veterinary Medicine, Ss. Cyril and Methodius University, Skopje, North Macedonia; 9 Roslin Institute and Royal (Dick) School of Veterinary Studies, University of Edinburgh, Midlothian, Scotland, UK; 10 Estonian University of Life Sciences, Tartu, Estonia; 11 Department of Animal Production, Faculty of Agriculture and Environment, Agricultural University ofTirana, Tirana, Albania; 12 College of Animal Science and Technology, China Agricultural University, Beijing, China; 13 Biotechical Faculty, Podgorica, Montenegro; 14 Faculty of Agriculture and Natural Resources, Arak University, Arak, Iran; 15 Department of Genetics, Physical Anthropology and Animal Physiology, University of Basque Country, Leioa, Spain; 16 Institute of Animal Husbandry, Belgrade-Zemun, Belgrade, Serbia; 17 Department of Animal Science, Biotechnical Faculty, University of Ljubljana, Ljubljana, Slovenia; 18 State Veterinary Institute Jihlava, Jihlava, Czech Republic; 19 https://lasig.epfl.ch/research/projects/projets-econogene/; 20 https://www.sheephapmap.org; 21 Faculty of Veterinary Medicine, Utrecht University, Utrecht, The Netherlands

## Abstract

**Background:**

In the Neolithic, domestic sheep migrated into Europe and subsequently spread in westerly and northwesterly directions. Reconstruction of these migrations and subsequent genetic events requires a more detailed characterization of the current phylogeographic differentiation.

**Results:**

We collected 50 K single nucleotide polymorphism (SNP) profiles of Balkan sheep that are currently found near the major Neolithic point of entry into Europe, and combined these data with published genotypes from southwest-Asian, Mediterranean, central-European and north-European sheep and from Asian and European mouflons. We detected clines, ancestral components and admixture by using variants of common analysis tools: geography-informative supervised principal component analysis (PCA), breed-specific admixture analysis, across-breed $f_{4}$ profiles and phylogenetic analysis of regional pools of breeds. The regional Balkan sheep populations exhibit considerable genetic overlap, but are clearly distinct from the breeds in surrounding regions. The Asian mouflon did not influence the differentiation of the European domestic sheep and is only distantly related to present-day sheep, including those from Iran where the mouflons were sampled. We demonstrate the occurrence, from southeast to northwest Europe, of a continuously increasing ancestral component of up to 20% contributed by the European mouflon, which is assumed to descend from the original Neolithic domesticates. The overall patterns indicate that the Balkan region and Italy served as post-domestication migration hubs, from which wool sheep reached Spain and north Italy with subsequent migrations northwards. The documented dispersal of Tarentine wool sheep during the Roman period may have been part of this process. Our results also reproduce the documented 18th century admixture of Spanish Merino sheep into several central-European breeds.

**Conclusions:**

Our results contribute to a better understanding of the events that have created the present diversity pattern, which is relevant for the management of the genetic resources represented by the European sheep population.

## Background

The domestic sheep descends from the wild Asian mouflon in southwest Asia and was, ca. 10.000 BCE (before the common era) together with goat, the first domestic livestock species [1]. As source of meat and milk, sheep has never reached the productivity of cattle and pigs, but has become the principal source of wool for textiles [2]. During the Roman era and the Middle Ages, the wool trade played a foremost role in the European economy [3–5]. By their toleration of extensive management, sheep have retained an important role in local economies of both the developing countries and the western world [6].

As for other livestock species, the post-domestication dispersal introduced sheep from southwest Asia to all inhabited continents [7, 8]. According to archaeological evidence, agriculture was introduced into Europe during the Neolithic Revolution following two routes, along the Mediterranean coasts and via the valley of the Danube [9–13], respectively. However, the resulting genetic clines may very well have been superseded by later events. For instance, a subsequent wave of migration is thought to have introduced into Europe the wool-type sheep, replacing most of the original hair-type sheep from which today’s feral European mouflon [14, 15] descends. A similar event has been the expansion around 3000 BCE of fat-tailed and fat-rumped sheep over central and southwest Asia and east Africa [16]. Roman written sources distinguish sheep producing coarse wool for carpets and fine wool sheep. The best wool sheep originated in south Italy and Greece [17, 18] and were exported to other parts of the Empire. In the late Middle Ages, the Spanish Merino breed was developed as producer of high-quality wool. Since the 16th century, it has been crossed into several French [19] and central-European breeds [20–22]. The use of several British breeds for upgrading northwest-European breeds, primarily as meat producers [14, 19], probably started later. In addition, it is plausible that wars, famines and epidemics have led to several, mostly undocumented mass eradications, after which flocks and herds had to be replenished by importations from elsewhere.

Differentiation of local sheep populations into breeds became more pronounced from the 18th century by the use of systematic breeding with well-defined objectives. The current sheep populations display a large diversity of local as well as transboundary breeds adapted to different environments and with different breeding objectives. A representative survey of sheep populations using the Illumina Ovine 50 K genome-wide single nucleotide polymorphism (SNP) panel [22] revealed a clear geographic differentiation, but also a high degree of historic admixture. Especially for Spanish breeds [22], this has been stimulated by seasonal transhumant migrations [23]. Current patterns of genetic diversity have been interpreted in historic and environmental terms, for example in Switzerland [24], mainland Italy [25, 26], Sicily [27], Belgium [28], France [19], the Pyrenean region [29], Spain [30], Greece [31], Wales [32], Russia [33], Nepal [34], China [35, 36], Iran [37], north Africa [38, 39], South Africa [40], Ethiopia [41, 42], New-Zealand [43] and the Carribean region [44] and Merino sheep generally [20]. Recurring observations and themes are the contrast between sheep with fat and normal tails [26, 33, 35, 41, 42], the influence of Merino sheep [19, 20, 33, 39] or other breeds [25, 44], the level of breed separation [27, 30, 32, 38, 39, 43] and the adaptation to the environment [34, 36, 37, 41, 45]. A comparison of European sheep with Asian and European mouflons [46] indicated an influence of European mouflons on domesticated sheep that has been relevant for adaptation.

In the above-mentioned studies, the authentic breeds from the Balkan countries, the major entry point of the Neolithic sheep into Europe [12], have been underrepresented. The Zackel type sheep with Pramenka and Ruda as subvarieties [47] are hardy sheep well adapted to extensive management in marginal areas.

**Fig. 1:**
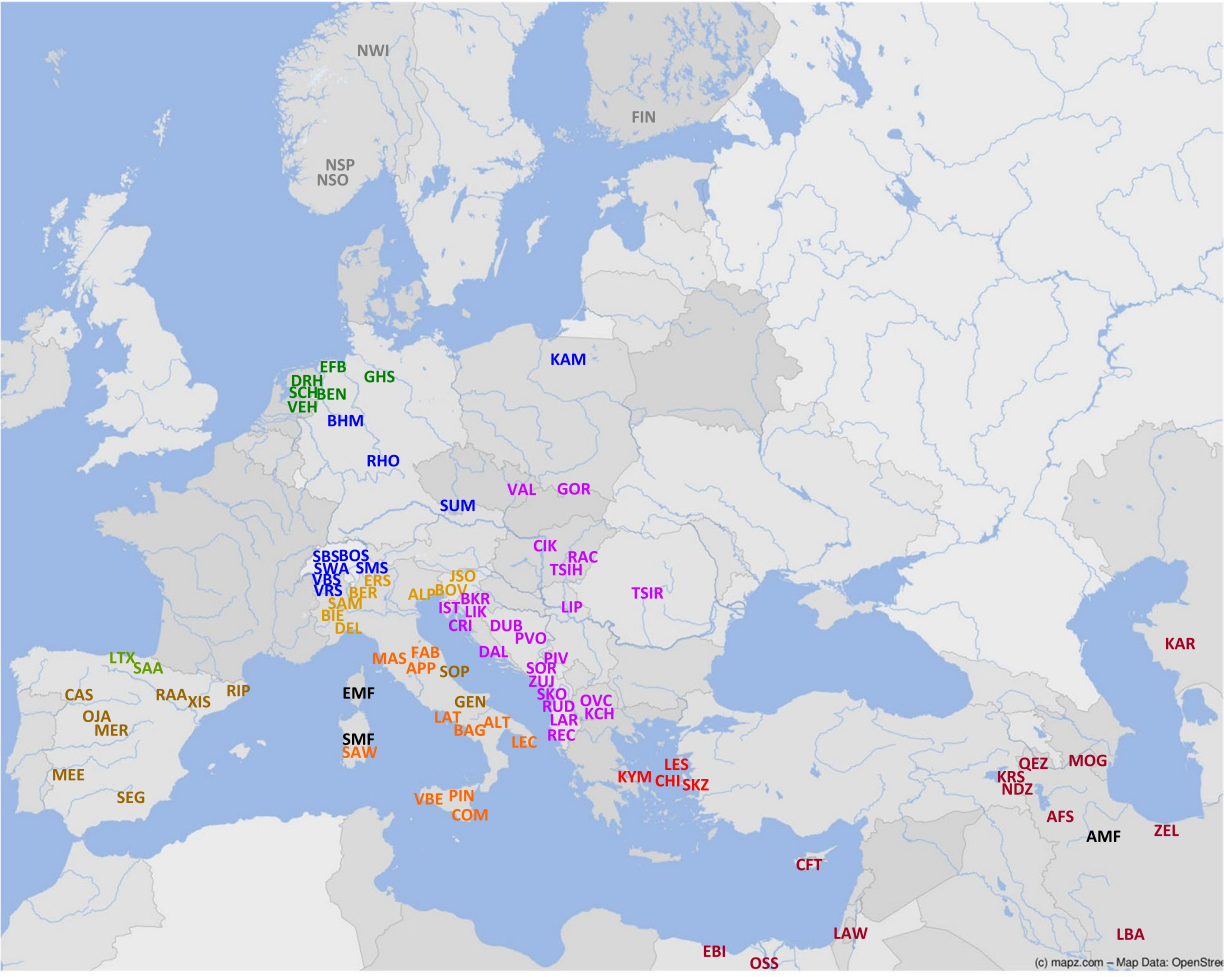
Breeds analyzed in this study. Breed codes: AFS, Afshari; ALP, Alpagota; ALT, Altamurana; AMF, Asian Mouflon; APP, Appenninica; BAG, Bagnolese; BEN, Bentheimer; BER, Bergamasca; BHM, Black-Headed Mutton; BIE, Biellese; BKR, Bela Krajina; BOS, Bundner Oberländer; BOV, Bovec; CAS, Castellana; CFT, Cyprus Fat-Tail; CHI, Chios; CIK, Cikta; COM, Comisana; CRI, Croatian Isles; DAL, Dalmatian; DEL, Delle Langhe; DRH, Drenthe Heath; DUB, Dubska; EBI, Egyptian Barki; EFB, East-Friesian Brown; EMF, European Mouflon; ERS, Engadine Red; FAB, Fabrianese; FIN, Finnsheep; GEN, Gentile di Puglia; GHS, German Heath; GOR, Polish Mountain; IST, Istrian; JSO, Jezersko-Solčava; KAM, Kamieniec; KAR, Karakul; KCH, Karakachanska; KRS, Karakas; KYM, Kymi; LAR, Lara; LAT, Laticauda; LAW, Local Awassi; LBA, Lori-Bakhtiari; LEC, Leccese; LES, Lesvos; LIK, Lika; LIP, Lipska; LTX, Latxa; MAS, Massese; MEE, Merino Estremadura; MER, Australian Merino; MOG, Moghani; NDZ, Norduz; NSO, Old Norwegian Spael; NSP, Spael-white; NWI, Norwegian White; OJA, Ojalada; OSS, Ossimi; OVC, Ovchepolean; PIN, Pinzirita; PIV, Pivska; PVO, Privorska; QEZ, Qezel; RAA, Rasa Aragonesa; RAC, Racka; REC, Recka; RHO, Rhön; RIP, Ripollesa; RUD, Ruda; SAA, Sasi-Ardi; SAM, Sambucana; SAW, Sardinian White; SBS, Swiss Black-Brown Mountain; SCH, Schoonebeker; SEG, Segurena; SKO, Shkodrane; SKZ, Sakiz; SMF, Sardinian Mouflon; SMS, Swiss Mirror; SOP, Sopravissana; SOR, Sora; SUM, Sumavska; SWA, Swiss White Alpine; TSIH, HungarianTsigaia; TSIR, RomanianTsigaia; VAL, Valachian; VBE, Valle del Belice; VBS, Valais Blacknose; VEH, Veluwe Heath; VRS, Valais Red Sheep; XIS, Xisqueta; ZEL, Zel; ZUJ, Zuja

In order to define more completely the historic relationships of European and southwest-Asian sheep and their phylogenetic relationships with the wild and feral populations, we generated 50 K SNP genotypes from a representative collection of Balkan sheep. These data were combined with available genotypes of south-, central- and north-European sheep and southwest Asian sheep (Fig. 1). By using variants of the most common modes of analysis, we identified the influence of the European mouflon and detected genetic clines, which could be historical witnesses of migration events. We have also used genotype data to test the historic evidence for the influence of the Spanish Merino sheep on several central European breeds.

## Methods

### Samples, genotypes, datasets

DNA was extracted from blood samples (see Additional file 1: Table S1) and used for genotyping on the Illumina Ovine 50 K SNP bead array as described previously [22, 25]. We used the Plink 1.9 program (http://www.cog-genomics.org/plink2) for data management and quality checks. Samples and markers with more than 10% missing genotypes were removed. The resulting data were combined with published genotypes for other breeds (see Additional file 1: Table S1). We used the Splitstree v4.14.6 software (https://software-ab.informatik.uni-tuebingen.de/download/splitstree4/welcome.html [48]) to construct Neighbour-Joining trees on the basis of allele-sharing distances (ASD) between individuals. We detected seven duplicate samples, whereas deviations from the expected clustering per breed in both novel and published data identified 16 outliers, i.e. samples for which the pure breed origin is questionable, presumably due to recent crossbreeding (AFS26, ALT15-18, BIE42, CAS03, IST38, LBA759571, LEC1, LEC2, LEC33, OJA10, SAM24, SAM49 and ZEL759420, all breed abbreviations are explained in the legend of Fig. 1). These samples together with those of declared F1 animals were discarded. In order to balance the composition of the dataset, we retained only the Australian Merino and the Spanish Estremadura populations from the available Merino samples. For most analyses, we used 25 or less individuals per breed. We retained 21,960 SNPs after linkage disequilibrium (LD)-pruning (Plink–indep-pairwise 50 50 0.03), which alleviates the ascertainment bias [49]. Additional file 2: Table S2 shows how datasets were tailored to the mode of analysis. From the dataset of [46], we retrieved genotypes from European mouflons sampled in Corsica, Sardinia and Hungary. For supervised admixture (v1.3.0) analysis [50], we assembled, based on the ASD NJ tree topology, a representive metapopulation (mEMF), consisting of 42 European mouflons sampled in Spain (2), Corsica (2), Hungary (8) and Sardinia (22 SMF-1 and 8 SMF-2 animals), respectively.

### Diversity and differentiation of breeds

Observed heterozygosities averaged per breed (*H*_o_) were calculated via Plink. Runs of homozygosity (ROH) longer than 1 Mb and containing more than 30 SNPs with an average density of more than one SNP/100 kb, a maximum gap between consecutive SNPs of 250 kb, and at most one missing and one heterozygous SNP, were calculated by using Plink as described previously [51]. We analyzed the differentiation of the Pramenka populations by means of the ASD Neighbour-Joining tree as described in the previous section. For the same purpose, data were filtered and phased haplotypes were inferred using the Shapeit v2.r900 software [52]. Shared haplotypes were identified by the program Chromopainter v2 and posterior distribution of clusters were visualized via the associated fineStructure v2 tree-building algorithm [53].

### Detection of clines

Principal component analysis (PCA) was performed with Plink, using a balanced dataset of less than six samples per breed for detection of genetic clines. To prevent bias arising from the large genetic distances between the most inbred sheep (East-Friesian Brown, Karakachanska and Valais Black-Nose) or the mouflons (AMF, EMF and SMF) and the other samples, we used the Plink option to calculate PCs for a subset of the individuals that are to be plotted, in this case all sheep except the EFB, KCH,VBS, AMF, EMF and SMF outliers. We refer to this procedure as supervised PCA (svPCA).

For the detection of geographic clines, we carried out a ‘geographic svPCA’ by calculating the PC only for the breeds living at the geographic extremes in the north (Norway, Finland), southwest (Spain) and southeast (southwest Asia, Egypt). We compared the performance of this method with the results of spatial PCA (sPCA [54]), which also has been designed for the detection of genetic clines, while using all three available triangulations (Delaunay, Gabriel and Nearest Neighbor) for alternative approximations of geographic inter-breed distances.

Neighbor-net graphs of Reynolds’ distances between breeds or regional groups of breeds were constructed as described previously [22].

### Ancestry and introgression

Model-based clustering using genome-wide SNPs was performed as implemented in the software Admixture v. 1.22 [50]. Admixture was further analyzed by three methods: model-based clustering with ancestry-informative markers (AIM [55]); calculation of $f_{4}$ statistics; and TreeMix tree constructions. The first two methods were used for detecting the influence of AMF, EMF and Merino, respectively. For this purpose, we assembled four metapopulations:

- mEMF, consisting of 42 mouflons that were less inbred than our Spanish EMF sample (2 Corsican, 8 Hungarian and 30 Sardinian mouflons [46] and 2 Spanish mouflons (EMF) with similar degree of inbreeding);
- PRMS, consisting of 28 southern Pramenka sheep (5 LAR, 5 OVC, 6 PIV, 2 REC, 5 RUD and 5 SOR) as proxy of the phylogenetic root of the European sheep;
- mMER, 26 Merino sheep (13 MER + 13 MEE);
- nMER, consisting of 127 non-Merino Iberian samples (21 CAS, 23 OJA, 27 RAA, 22 RIP, 12 RAA and 22 XIS).

The use of panels of AIM for model-based clustering as implemented in Structure [56] has been demonstrated in [35] for fat-tailed sheep and is denoted here as breed-specific admixture analysis (BSAA). AIM were selected based on their *F*_ST_ values calculated via Plink. Specifically, we selected 358 AMF-specific AIM while avoiding AMF-mEMF cross-specificity (see Additional file 3: Table S3A) by using the following thresholds: *F*_ST_ (AMF-PRMS) > 0.5, *F*_ST_ (mEMF-AMF) > 0.5 and *F*_ST_ (mEMF-PRMS) < 0.1. Likewise, 334 EMF-specific SNPs (see Additional file 3: Table S3B) were selected by using the thresholds: *F*_ST_ (mEMF-PRMS) > 0.6, *F*_ST_ (mEMF-AMF) > 0.6 and *F*_ST_ (AMF-PRMS < 0.1). For testing Merino admixture, 606 SNPs (see Additional file 3: Table S3C) were selected by using the threshold *F*_ST_ (mMER-MER) > 0.13. Thus, the AIM panels defined were used for Structure analysis with 15,000 burn-in steps and 35,000 iterations at different k values. Q-values from the run with the lowest *k* value showing a breed-specific signal were plotted.

The $f_{4}$ statistic uses allele frequencies from four populations: a source and a recipient of ancestry (often via introgression) to be tested and for each a related control population free of the ancestry to be tested. The ancestry of the source in the recipient generates a significant correlation between the shifts in allele frequency between the source and its control (for instance, an outgroup) and between the recipient and its control, respectively [57]. The influence of Asian and European mouflons, respectively, relative to PRMS was detected by:

$f_{4} = \left( {f_{\text{AMF}} - f_{\text{mEMF}} } \right)\left( {f_{{ {\text{PRMS}}}} - f_{{ {\text{X}}}} } \right)$, averaged across 21,960 SNPs, where $f$ is the allele frequency of one of the two SNP alleles in the indicated breed and ${\text{X}}$ is the recipient test breed. The results were normalized by the ratio:

$f_{{4{\text{n}}}} = f_{4} / \left( {f_{\text{AMF}} - f_{\text{mEMF}} } \right)\left( {f_{{ {\text{PRMS}}}} - f_{{ {\text{X}}}} } \right),$ with the nominator and denominator averaged over 21,960 SNPs.

Positive and negative values indicate that, relative to PRMS, the influence of European mouflon is larger and smaller, respectively, than the influence of Asian mouflon. The confidence interval of $f_{{4{\text{n}}}}$ has been calculated as $f_{{4{\text{n}}}}$ plus 2 × its standard deviation parallel to $f_{{4{\text{n}}}}$ minus 2 × its standard deviation, corresponding to a *P* < 0.05 for $f_{{4{\text{n}}}}$ > 0 or < 0.

Likewise, the influence of Merino sheep on other breeds was inferred from positive values of

$$f_{{4{\text{n}}}} = \left( {f_{\text{nMER}} - f_{\text{mMER}} } \right)\left( {f_{{{\text{PRMS}}}} - f_{{{\text{X}}}} } \right) / \left( {f_{\text{nMER}} - f_{\text{mMER}} } \right)\left( {f_{{{\text{PRMS}}}} - f_{{{\text{mMER}}}}}\right),$$

with the nominator and denominator averaged over 21,960 SNPs.

Values are close to 1.0 for the Merino as test breed (1.05 for MEE and 0.94 for MER) and yield for the other breeds estimators of the degree of Merino introgression relative to PRMS.

TreeMix [58] was used to construct a maximum likelihood tree of 78 breeds or regional groups of breeds, to which 1 to 20 edges were added as indicators of admixture events. Likelihoods and proportions of variance explained were calculated by using the R Package OptM (B. Fitak, https://cran.r-project.org/web/packages/OptM).

## Results

### Breed diversity and differentiation

Our panel of sheep comprises several breeds from southeast Europe and surrounding areas: southwest Asia, Egypt, Italy, Spain and central and north Europe (Fig. 1) and (see Additional file 1: Table S1). The diversity as derived from the observed heterozygosity (*H*_o_) is highest in most of the eastern, southeast-European and Iberian breeds. *H*_o_ values are lower in most Italian, Swiss, Greek and the Asian and African Fat-tailed sheep and decreased further in the north-European breeds. The extremely low values for East-Friesian Brown, Karakachanska, Valais Blacknose and the European Mouflon are likely reflecting genetic isolation and/or population bottlenecks. However, whole-genome sequencing has shown that the nucleotide diversity of Asian mouflons is higher than that of domesticated sheep [59–61]. This indicates that the low *H*_o_ values for the mouflons reflect the ascertainment bias of the 50 K SNP panel, i.e. the diversity of the breeds that were not used to create the SNP panel is underestimated. This explanation is supported by a plot of *H*_o_ against the total ROH coverage (*F*_ROH_, [see Additional file 4: Table S4]), in which the latter value is expected to be less affected by the ascertainment bias than *H*_o_. For most breeds, this plot (see Additional file 5: Figure S1) shows the expected inverse linear relationship [62], but H_o_ values for Asian mouflon and, to a lesser degree, Sardinian mouflon, fat-tailed sheep and Balkan breeds are relatively low.

We tested the breed-level differentiation by visualizing ASD between individuals in Neighbour-Joining trees. Most breeds are well separated with only a nesting of Norwegian Spæl White within Original Norwegian Spæl and a good correlation of within-breed distances and *H*_o_ (not shown). However, there is a high degree of intermingling of the Pramenka breeds (see Additional file 6: Figure S2).

A fineStructure plot based on haplotype sharing (see Additional file 7: Figure S3) confirms the incomplete breed differentiation of Pramenka sheep with the intermingling of Dalmatian, Lara, Lipska, Ovchepolean and Romanian Tsigaia as also revealed by the ASD tree. Diagonal clusters of related sheep correspond to the most distinct breeds or to regional combinations of Pramenka breeds (Hungarian Racka, Slovenian Bela Krajina, Croatian Lika, Bosnian Dubska and Privorska; Montenegrin Pivska and Sora; and Serbian Lipska and north-Macedonian Ovchepolean, respectively).

### Coordination analysis

**Fig. 2:**
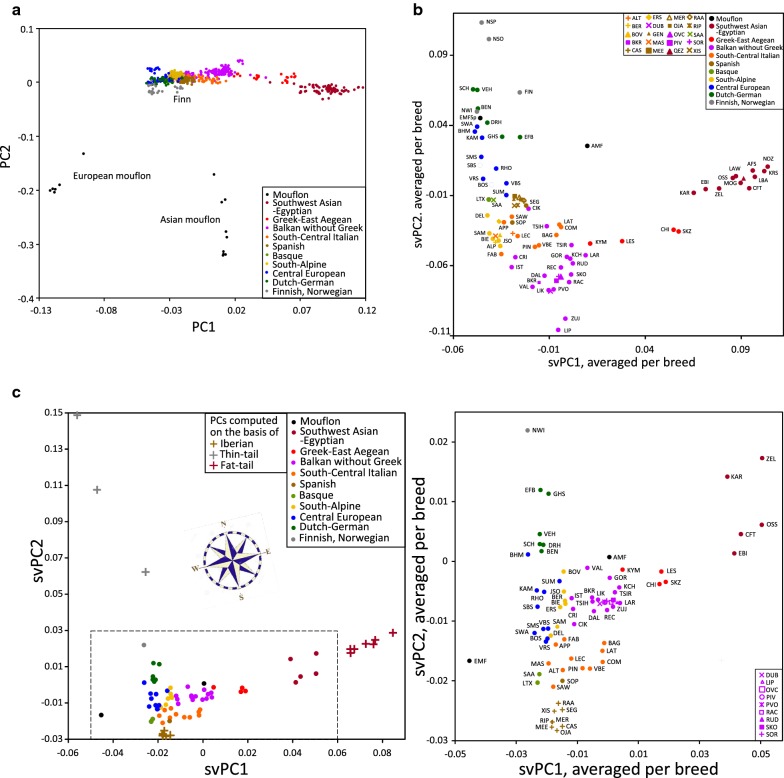
PCA analysis (breed codes are as in Fig. 1). **a** Plots of 546 domestic sheep (≤ 6 animals per breed). We checked that removal of either European or Asian mouflons did not change the pattern of the other individuals. Finn sheep are relative to other Nordic breeds shifted toward the fat-tailed sheep. **b** Supervised PCA of 1477 animals in which the PC values were calculated based on 507 domestic animals (≤ 6 animals per breed; without EFB, KCH, VBN, AMF, EMF or SMF) and have been averaged per breed. See Additional file 9: Figure S5 for svPC3 vs. svPV1 and see Additional file 8: Figure S4 right panels, for the corresponding plots of individuals. **c** Left panel: supervised PCA of 1477 animals in which the PC values (svPC1, svPC2), averaged per breed, were calculated based on the indicated fat-tailed, Nordic and Spanish sheeps. Right panel: magnification of the area indicated by the dotted line in the left panel. See Additional file 11: Figure S7 for the corresponding plot of individuals

Figure 2a shows a PCA plot of domesticated sheep and European and Asian mouflons. PC1 shows an east–west cline of domesticated sheep between the Iranian fat-tailed sheep and the north-European thin-tailed sheep. Remarkably, this trend is at the west end extrapolated towards the European mouflon. PC2 separates mouflons and domestic sheep. Asian mouflons are almost equidistant to all other sheep, including the northwest-Iranian domestic sheep from the region where the Asian mouflons were sampled.

In a PCA of domesticated sheep (see Additional file 8: Figure S4 left panels), PC1 again shows the east-to-west cline, whereas PC2 and PC3 are disproportionally influenced by East-Friesian Brown (EFB), Karakachanska (KCH) and Valais Blacknose (VBS), the three breeds with low *H*_o_. This is remedied by supervised PCA (svPCA) in which we computed the principal components for the domesticated sheep without these three breeds (see Additional file 8: Figure S4 right panels). The svPCA interpolates these breeds close to other sheep from the same region, demonstrating that this approach extends the usefulness of PCA to populations with extremely low diversity.

In Fig. 2b, the svPC values have been averaged over the breeds. In this plot, svPC1 shows an east–west cline that is particularly strong for the Greek breeds. SvPC2 values show a cline from the Balkan region along the Mediterranean coast and as well as a cline south to north. SvPC3 emphasizes the contrast of northern and southwest Europe (see Additional file 9: Figure S5).

Spatial PCA (sPCA) combines genetic and geographic information in order to optimize the detection of geographical trends [54], with a choice of three methods of triangulation to calculate spatial distances between breeds. However, with our dataset, the three methods of triangulation in sPCA (see Additional file 10: Figure S6) generated essentially the same pattern as PCA (see Additional file 8: Figure S4 right panels).

Additional file 11: Figure S7 shows an alternative way to introduce geographical information: supervised PCA in which the components are controlled by the geographical extremes: the fat-tailed breeds from southwest Asia, the southwest-European breeds from Spain and the northernmost breeds from Norway and Finland. Then, the other sheep and the mouflons are interpolated between these extremes without using their mutual distances. Compared to Fig. 2 and Additional file 8: Figure S4, averaging the geographic svPC values per breed (Fig. 2c) emphasizes a central position of north Italy between the Balkans, central and south Italy, Spain and central Europe, indicating both an east–west and a north–south cline. The plot of individuals (see Additional file 11: Figure S7) may suggest direct contact between the Balkan and south-Italian sheep, but this is mediated by the Bagnolese and Laticauda sheep, which are known to have been influenced by north-African fat-tailed Barbary sheep [26]. In Fig. 2c, Asian mouflons are interpolated within the Balkan sheep, whereas in agreement with Fig. 2a, European mouflons are extrapolated to an extreme left position.

### Phylogenetic analysis

Because of the incomplete genetic differentiation of the Pramenka breeds and their small sample size, we combined neighboring populations and other closely related breeds in regional clusters, as indicated in Additional file 12: Table S5A. Visualization of Reynolds’ genetic distances in a Neighbor-network phylogenetic graph (see Additional file 13: Figure S8) generates an east–west axis with a separate branching-off of fat-tailed, Balkan and the other European breeds, following closely the cline highlighted by the geographical svPC1 in Fig. 2c. The long terminal distances of several Balkan breeds reflect the increase of the Reynolds’ distances by small samples sizes, but also the low heterozygosity of Cypriotic fat-tailed (CFT), East-Aegean Chios and Sakiz (CHI, SKZ), Karakachanska (KCH) and Valachian (VAL). Among the Pramenka breeds, Hungarian Cikta, Istrian and Croatian Isles (CIK, IST, CRI) are the closest to the other European breeds. Breeds from the same country are in the same area of the network, but Polish Kamieniec (KAM) clusters with the Swiss Alpine and Swiss Black-Brown Mountain (SWA, SBS).

**Fig. 3:**
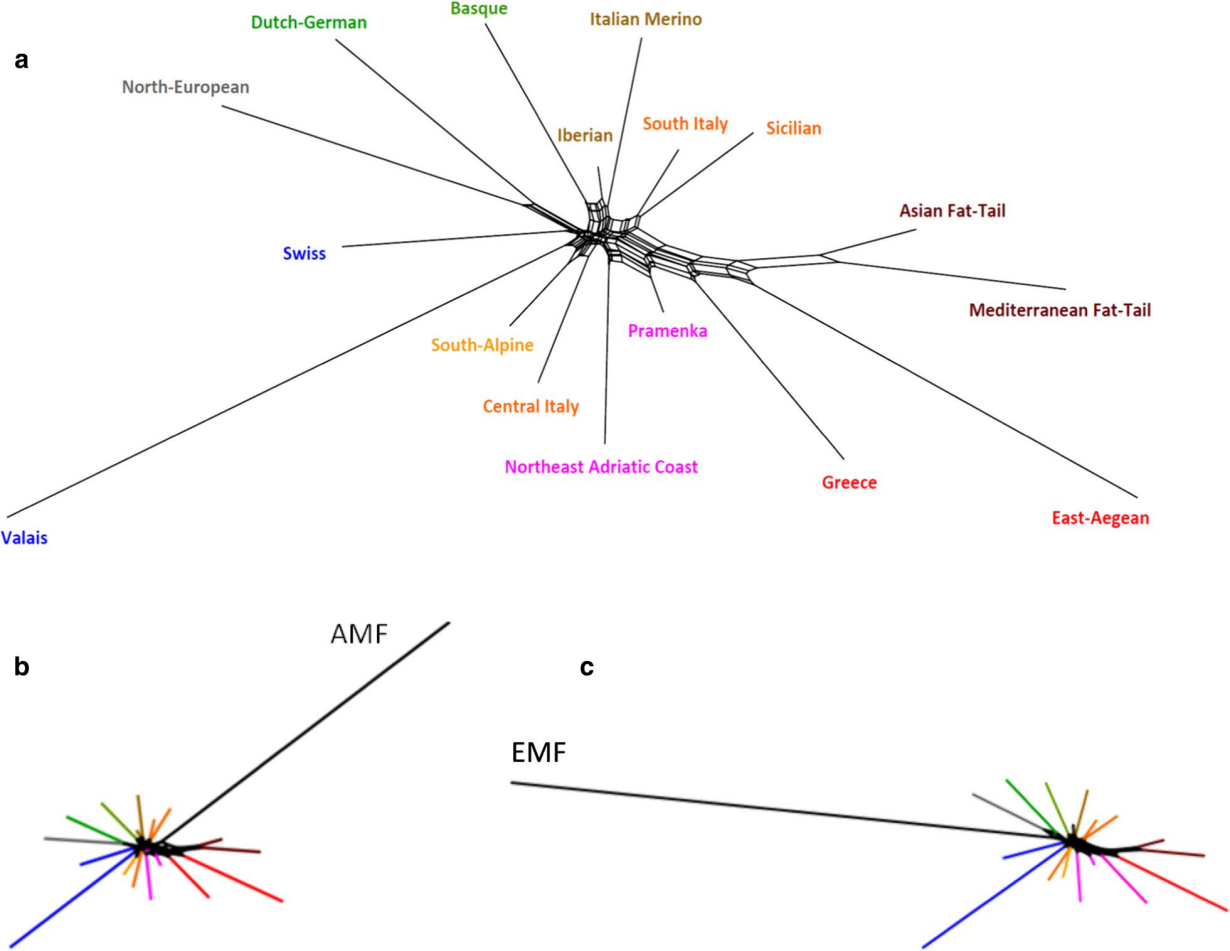
**a** Neighbor-net graph of Reynolds’ distances between regional groups of breeds (see Additional file 12: Table S5B). **b**, **c** Patterns obtained by including AMF and EMF, respectively (see Additional file 14: Figure S9)

A further grouping of breeds according to their relatedness and geography (see Additional file 12: Table S5B) improves the resolution of the European sheep (Fig. 3a). In agreement with the PCA, the topology of the tree suggests that gene flow from the Balkan region was followed by a radiation to Spain and central and north Europe. Adding Asian Mouflon (AMF, Fig. 3b) and European Mouflon (EMF, Fig. 3c) to this network suggests their affinity to southeast- and north-European sheep, respectively, which is in agreement with the PCA plot of Fig. 2a. As shown in Additional file 14: Figure S9, the differential affinities of AMF and EMF to domesticated sheep can also be visualized in the same network after resolving the reticulation caused by the affinity of AMF and EMF.

### Model-based clustering

**Fig. 4:**
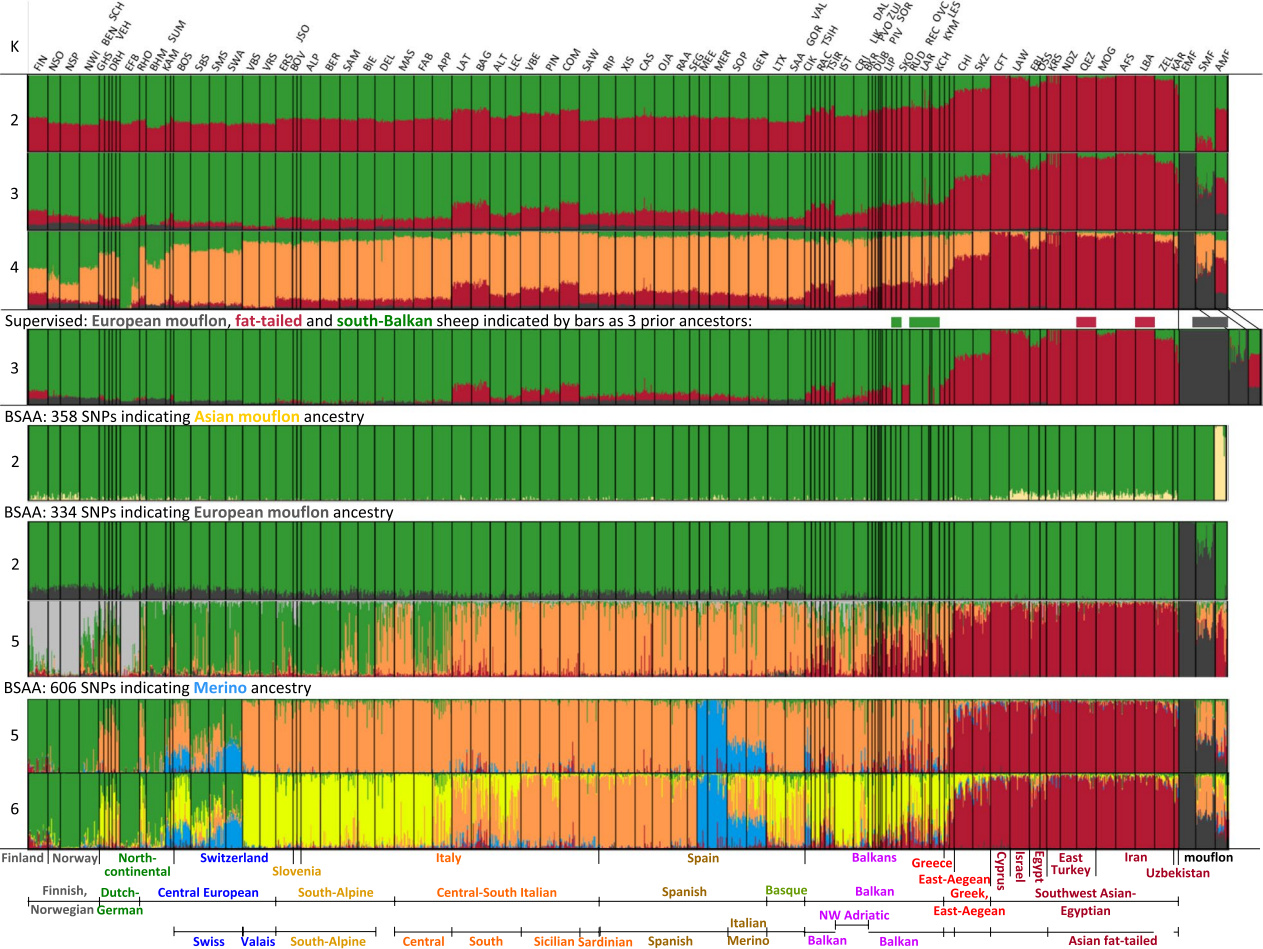
Model-based clustering generated by the program Admixture (first four bar plots) or breed-specific admixture analysis (BSAA) generated by Structure (other plots). Admixture has been run unsupervised (first three plots) or supervised by prior information for three clusters as shown by the thick colored bars. In this analysis, the dataset has been supplemented with additional mouflon samples [46]. For the selection of SNPs for BSAA, see Methods. Regions and countries have been indicated below the plots; in this plot ‘Balkan’ does not include Greek breeds

As observed previously [20, 63], clustering by the admixture program (Fig. 4) did not reproduce the differentiation of sheep from different regions observed with coordination or phylogenetic analysis, which is in contrast to the admixture patterns for European goats or cattle [64, 65]. With the number of clusters (*k*) set at 2, 3 or 4, one cluster consistently corresponds to the fat-tailed sheep. At *k* = 3, another cluster corresponds to the mouflons, and *k* = 4 generates an incomplete split-off of northern Europe. Higher *k* values (not shown) generated clusters corresponding to one or two breeds.

These patterns suggest a fat-tailed influence on Finn sheep and on several Mediterranean and Balkan breeds. The strongest signals were observed for the Italian fat-tailed Laticauda and Bagnolese and for the Sicilian, Hungarian and most of the Pramenka breeds. In addition, a low but consistent signal of mouflon ancestry shows an increase from southeast to north Europe. This is consistent with Fig. 1 and becomes more pronounced if the Admixture program is run in the supervised mode (Fig. 4). This plot also suggests introgression of domesticated into the Sardinian mouflon (SMF) population as reported previously [46].

### Admixture

**Fig. 5:**
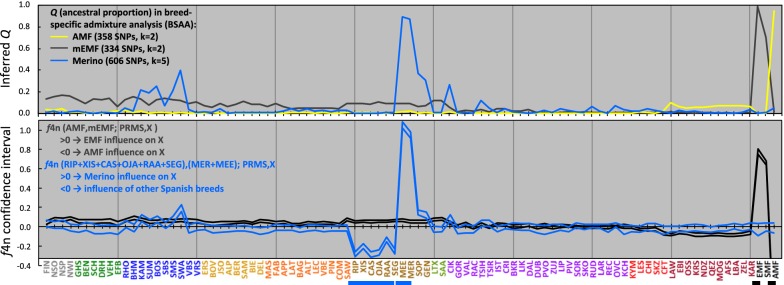
Top panel: Q values indicating ancestry of AMF, EMF and Merino (MER + MEE), respectively, as inferred from the corresponding BSAA runs shown in Fig. 4. Bottom panel: Scan of indicated *f*4n values over the 93-breed panel. The metapopulation PRMS has been defined in Methods. The blue and black line below the axis labels indicate the (groups of) breeds for which ancestry can be inferred from positive or negative *f*4n values

The signal of mouflon ancestry in the admixture patterns does not differentiate between European and Asian mouflons. Therefore, following the breed-specific admixture analysis (BSAA) procedure as detailed in Methods, we selected ancestry-informative markers (AIM) that differentiate between AMF and EMF ancestry. Model based clustering by using the Structure program (Fig. 4, 5th and 6th bar plot, Fig. 5, top panel) highlights different patterns for Asian and European mouflon ancestry. AMF ancestry is weak and is observed only in northern and fat-tailed breeds. EMF ancestry was inferred for most European breeds with the highest signals in north European, Swiss and Basque sheep.

Figure 4 (last two plots) and Fig. 5 (top panel) show a BSAA for Merino introgression. In addition to the expected introgression in the Italian Merino breeds Sopravissana and Gentile di Puglia, there are clear signals in Czech Sumavska, Polish Kamieniec, Swiss Bundner Oberländer, Mirror and Alpine, Slovenian Jezersko-Solčava, Hungarian Cikta and Tsigaia, Albanian Ruda and north-Macedonian Ovchepolean.

The BSAA signals for AMF, EMF and Merino are confirmed by a normalized $f_{4}$ analysis, which detects introgression via correlation of allele frequencies (Fig. 5, bottom panel). This generates weaker signals, but yields plausible quantitative estimates of the degree of admixture, which for the AMF and EMF ancestries are calculated relative to the southern Pramenka (PRMS) sheep.

The TreeMix program offers a sophisticated and currently favored approach to combine phylogenetic trees with identification of admixture events [58]. The tree generated without assuming migrations (*m* = 0) (see Additional file 15: Figure S10) agrees with the Neighbor-net graph (see Additional file 14: Figure S9), but joins the feral EMF and SMF mouflons to the Nordic and Dutch-German Heath breeds as in Additional file 14: Figures S9D and S9E. Several but not all gene flows indicated by the colored line in the *m* = 6, 10 or 20 patterns are consistent with the admixtures found by BSAA and *f*4 analysis. A more extensive description of theTreeMix pattern is in Additional file 15: Figure S10.

## Discussion

### Scope of this study

In order to identify historic and prehistoric gene flows and admixture events, we analyzed genotypes of several Balkan sheep breeds together with those from southwest-Asian, Mediterranean, central-European and north-European populations. Our samples cover the area through which sheep were originally introduced into Europe, and initiated their dispersal all over the continent. We have tested several variants of existing modes of analysis, which may have a more general applicability. A geographically supervised PCA appeared to be more effective to detect genetic clines than the spatial PCA [54], while the admixtures identified consistently by breed-specific admixture analysis and $f_{4}$ analysis could be only partially reproduced by the TreeMix algorithm. For more detailed methodological considerations, please refer to Additional file 16.

### Mouflons and domestic sheep

The current Asian mouflons that were sampled in northwest Iran near the putative southwest-Asian sites of domestication are only distantly related to present-day sheep (Figs. 2a, 3b) [19, 59, 66]. Thus, the genetic distances generated a network (Fig. 3) that places Asian mouflons close to the center of the network near the Pramenka, Greek and fat-tailed sheep, but without any affinity to any of the present-day breeds, including the fat-tailed breeds currently found in the area which was the original domestication centre. We tested whether this could be an artefact of the ascertainment bias, by which the diversity of Asian mouflons is known to be severely underestimated [59, 60]. However, we obtained the same topology when we selected SNPs with a high minor allele frequency (> 0.39) in the Asian mouflon [49]. This suggests that a SNP panel with additional mouflon SNPs, for instance as obtained by whole-genome sequencing, would have revealed similar relative affinities of mouflon to the domesticated sheep [67].

A plausible explanation for the divergence of Asian mouflons and domestic sheep is provided by a recent analysis of Y-chromosomal variation (unpublished results), which showed for a panel of Asian mouflons, as our panel sampled in Iran, a Y-chromosomal haplotype that does not resemble any domestic haplotype, but is closely similar to the haplotype of the cross-fertile urial (*Ovis vignei*). This may indicate that at least these mouflons have diverged by urial introgression from the mouflon population that was the ancestral source of the domesticated sheep.

The earliest domesticated sheep in Europe were the ancestors of today’s feral European mouflons [16] and have been replaced in agriculture by wool sheep. Traces of European mouflons in domesticated sheep increase from southeast to northwest (Figs. 2a, 3, 4). This is in agreement with [15], but genome-wide markers now yield estimates of the relative degrees of mouflon ancestry. Barbato et al. [46] proposed that this mouflon component contributed to the environmental adaptation of domesticated sheep.

### The origin of European sheep

The replacement of the first domesticated sheep by wool sheep may have already started by 4000 BCE [8, 14]. In a similar, rather later process, fat-tailed sheep became predominant in many locations of Asia [14]. Remarkably, the thin-tailed Zel sheep is found to be in the same genetic cluster as the fat-tailed Iranian sheep whereas the fat-tailed Italian Laticauda [25] is related to other breeds in central Italy. This implies that the tail phenotype is encoded by a limited number of genes [36, 68–72]. This is in sharp contrast to, for example, the deep-rooted split of taurine and zebu cattle.

In spite of the long history and economic importance of the production of wool, several sheep with a coarse fleece have been maintained over time. At the time of the Roman Empire, the best quality wool was that from the ‘Tarentine’ sheep, also known as ‘Greek’ sheep [18]. Sheep from Italy, presumably those with fine-wool, were exported to other parts of the Roman empire [3]. However, the process was not complete and European coarse and fine-wooled sheep still have overlapping and fragmented distributions. The present Iberian coarse-wool Churra and the fine-wool Merino are even close relatives [22]. We propose that this phenotypic differentiation reflects the opposing and varying effects of human selection for fine wool production and environmental adaptation favoring the more natural coarse wool. As for the tail traits, wool quality is controlled by a restricted number of genes [59, 73, 74].

As found previously for cattle and goats [64, 65], coordination, phylogenetic and clustering analyses (Figs. 2, 3, and 4) consistently show that regional origin is the primary determinant of genetic differentiation. This has generated several clusters of related breeds. Greek breeds are intermediate between fat-tailed Asian and European sheep. The several incompletely differentiated Zackel breeds, most of which were kept in the former Ottoman Empire, form one of the coherent breed clusters. In agreement with a history of mixed German (‘Zaupel’)-Hungarian origin, Cikta is intermediate between the Zackel and central European sheep. Similarly, Delle Langhe of north Italy is not closely related to other north Italian breeds (see Additional file 13: Figure S8) and may have been influenced by breeds not included in our dataset.

Correlation of genetic distances of breeds or clusters with corresponding geographic distances indicate genetic clines. Plausibly, the clines detected by svPCA (Fig. 2b, c) correspond to the expansion of wool sheep, which may have overruled the earlier clines formed during the introduction of farming along the Mediterranean and Danubian routes [9–13]. If we assume that the maintenance of steep clines indicate a relatively slow gene flow, the PCA plots and networks indicate that Greek breeds acted as a barrier between Asian and European breeds. The spatial svPCA patterns (Fig. 2c) also suggest a gene flow from the Balkans into north Italy. This intersects with the direction of a gene flow from north Italy to central and south Italy and then to Spain and with a third gene flow from north Italy towards central and north Europe. Remarkably, there seems to have been no direct flow from the northernmost Zackel sheep, Czech Valachian and Polish Mountain, to sheep of north Europe.

In addition, the genetic proximity of Zackel, central and south-Italian and Spanish breeds (Figs. 2b and 3) suggests a migration route along the Mediterranean littoral from the Balkans to Spain via the Italian peninsula. This is not in contradiction with the geographic svPCA (Fig. 2c), because the gene flows corresponding to the Mediterranean east–west movement may very well have influenced the allele frequencies in other SNP panels than those selected in the geographic svPCA. Plausibly, in addition to the Neolithic expansions of agriculture following the Mediterranean and Danubian routes [9–13], the expansion of the Tarentine sheep over the Roman Empire contributed to both the Mediterrenean migrations and to the northward migration from Italy into central and north Europe.

The Admixture and Structure pattern (Fig. 4) shows a minor fat-tail component in Finn sheep, which is consistent with the PCA plots (Fig. 2a, b) and suggests an influence of Asian sheep.

A more recent event is the documented dispersal of Merino sheep since the 17th century [21]. As expected, there is a large Merino component in the Italian Merino-type Sopravissana and Gentile di Puglia. In addition, we found clear Merino signals in several central-European breeds. Although these breeds do not have a Merino-type wool, these introgressions are entirely in agreement with the documented upgrading in the 17th and 18th century. The strongest signal is found in the Swiss Alpine, whereas the genome of its close relative the Polish KAM also has a substantial Merino component. The Swiss breeds SWA, SBS and SMS were putatively influenced by British sheep as well [22] as inferred from their high frequency of the Y-chromosomal oY1.1 *G* allele, which is fixed in several British breeds [75]. KAM is known to have been influenced by Romney and Texel influence [47]. Notably, breed histories of SWA and KAM do not mention their close relationship or their Merino ancestry [24, 47], which demonstrates the power of genome-wide DNA analysis for uncovering hidden relationships and admixture events.

Our breed panel did not include French or British breeds. Kijas et al. [22] showed that English and Scottish breeds are well differentiated from other European breeds. Rochus et al. [19] found, for north-French breeds an affinity to English breeds known to have been used for upgrading, and for south-French sheep affinities to Italian and Spanish breeds. A more complete sampling of British, north-continental and Nordic breeds would enable a dissection of gene flows in northern Europe and a documentation of the consequences of the frequent upgrading of north- and central-continental breeds by English rams.

## Conclusions

Our study of the Balkan breeds is a crucial addition to the panel of breeds analyzed so far with the genome-wide SNP arrays. We propose that both the Balkans and Italy were hub regions from which sheep dispersed over the rest of Europe. We demonstrate that the use of various variants of PCA, phylogenetic analysis and model-based clustering, emphasizes combinations of SNPs that are informative for genetic events. Our results consistently suggest a number of prehistoric and historic gene flows. All this contributes to a better understanding of the genetic background of cosmopolitan and local sheep breeds, serving as templates of environmental adaptation and human selection.

## Supplementary information

**Additional file 1: Table S1.** Sheep breeds analyzed in this study [16, 18, 20, 22, 31, 35, 47, 59, 76]. Colors indicate genetic clusters. Boxes indicate breeds combined in the 78-breed panel.

**Additional file 2: Table S2.** Datasets used for analysis.

**Additional file 3: Table S3.** A 358 SNPs informative for Asian Mouflon ancestry used in BSAA. Only SNPs were considered with > 40 out of 42, > 55 out of 57 and > 67 out of 69 non-missing allele frequencies used for the pairwise AFM-PRMS, AMF-EMFM and EMFM-PRMS *F*_ST_ calculations, respectively. B 334 SNPs informative for Asian Mouflon ancestry used in BSAA. Only SNPs were considered with > 67 out of 69, > 55 out of 57 and > 40 out of 42 non-missing allele frequencies used for the pairwise EMFM-PRMS, AMF-EMFM and AFM-PRMS *F*_ST_ calculations, respectively. C 606 SNPs informative for Merino ancestry used in BSAA. Only SNPs were considered without missing allele frequencies used for the pairwise Merino-(Iberian non-Merino) *F*_ST_ calculations.

**Additional file 4: Table S4.** ROH statistic per individual or averaged per breed. *F*_ROH_ is the total length of the ROH divided by the total length of the sheep autosomes (2452.06 Mb).

**Additional file 5: Figure S1.** Inverse linear relationship of observed heterozygosity and the total ROH coverage *F*_ROH_, showing relatively low heterozygosity values for AMF, SMF and fat-tailed sheep.

**Additional file 6: Figure S2.** Neighbour-joining tree visualizing the allele-sharing distances of the Balkan sheep (see Fig. 1) or (see Additional file 1: Table S1) for the breed codes). Breeds that are dispersed over different branches of the tree are indicated by colored lines.

**Additional file 7: Figure S3.** FineStructure clustering of eastern and southeastern European sheep breeds. The color of each bin in the matrix indicates the number of “genomic chunks” copied from a donor (columns) to a recipient individual (rows).

**Additional file 8: Figure S4.** Left panels: normal PCA plots of 525 sheep (≤ 6 per breed) including the inbred EFB, KCH, VBS. Right panels: supervised PCA of 546 sheep, including three mouflon populations, in which EFB, KCH and VBS as well as the mouflons have been excluded for calculation of the principal components (svPC1, svPC2 and svPC3).

**Additional file 9: Figure S5.** Supervised PCA of 546 animals as in Fig. 2b, showing svPC1 vs. svPC3 averaged per breed.

**Additional file 10: Figure S6.** Spatial PCA of 507 domestic sheep without EFB, KCH and VBS, which were found to dominate the sPC2 and sPC3 just as for in the normal PCA (Additional file 8: Figure S4 left panels). The three methods of triangulation, indicated above the plots, give essentially the same results, which are similar to the supervised PCA pattern (see Additional file 8: Figure S4 right panels).

**Additional file 11: Figure S7.** Supervised PCA of 546 animals in which the PC (svPC1, svPC2) were calculated based on the indicated fat-tailed, Nordic and Spanish sheep.

**Additional file 12: Table S5.** Grouping of breeds for calculation of genetic distances. (A) Regional monophyletic groups of breeds for the Neighbor-net graph in Additional file 13: Figure S8. (B) 17 Regional groups of related breeds for the Neighbor-net graphs in Fig. 3 and Additional file 14: Figure S9.

**Additional file 13: Figure S8.** Neighbor-net graph of Reynolds’ distances between breeds or regional combinations of closely related breeds (see Additional file 12: Table S5).

**Additional file 14: Figure S9.** Neighbor-net graphs of 17 regional groups of breeds (Additional file 12 B) with (A) AMF, (B) EMF, (C, D) both AMF and EMF; (D, E) pattern obtained by increasing the AMF-EFM distance in order to suppress the EMF-AMF clustering and to show different affinities of EMF and AMF for European domestic sheep.

**Additional file 15: Figure S10.** TreeMix trees without and with 6, 10 and 20 migrations and plots of the proportions of the variance explained (f-indices) and likelihoods at different *m* values. Coloured lines indicate inferred migrations with a weight according to the color scale.

**Additional file 16.** Methodological comparisons and considerations [22, 50, 53, 54, 56, 64, 65, 77].

## Data Availability

The datasets supporting the conclusions of this article are available in the Figshare repository via 10.23644/uu.8947346. Data for the Corsican, Hungarian, and Sardinian European mouflon are from [46].
