# Supplementary material for: On the origin of European sheep as revealed by the diversity of the Balkan breeds and by optimizing population-genetic analysis tools
Source: Genet Sel Evol. 2020 May 14;52:25. doi: 10.1186/s12711-020-00545-7 (PMC7227234; doi:10.1186/s12711-020-00545-7)
Supplement: Supplementary file 5 — Additional file 5: Figure S1. Inverse linear relationship of observed heterozygosity and the total ROH coverage FROH, showing relatively low heterozygosity values for AMF, SMF and fat-tailed sheep. [file 12711_2020_545_MOESM5_ESM.docx]

**Additional file 5 Figure S1**. Inverse linear relationship of observed heterozygosity and the total ROH coverage *F*_ROH_, showing relative low heterozygosity values for AMF, SMF and fat-tailed sheep.
