# Supplementary material for: On the origin of European sheep as revealed by the diversity of the Balkan breeds and by optimizing population-genetic analysis tools
Source: Genet Sel Evol. 2020 May 14;52:25. doi: 10.1186/s12711-020-00545-7 (PMC7227234; doi:10.1186/s12711-020-00545-7)
Supplement: Supplementary file 6 — Additional file 6: Figure S2. Neighbour-joining tree visualizing the allele-sharing distances of the Balkan sheep (see Fig. 1) or (see Additional file 1: Table S1) for the breed codes). Breeds that are dispersed over different branches of the tree are indicated by colored lines. [file 12711_2020_545_MOESM6_ESM.pptx]

## Slide 1
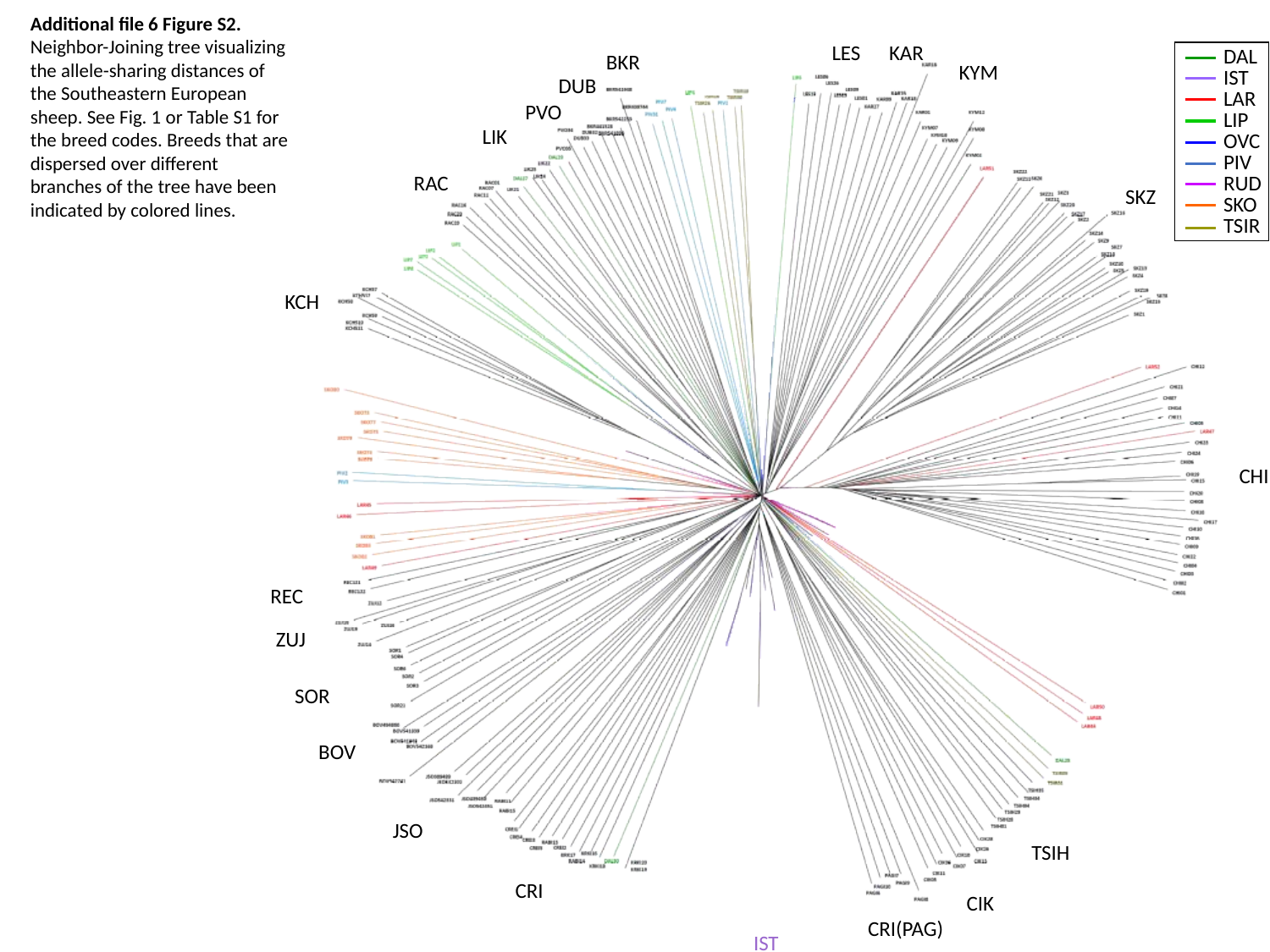

Additional file 6 Figure S2. Neighbor-Joining tree visualizing the allele-sharing distances of the Southeastern European sheep. See Fig. 1 or Table S1 for the breed codes. Breeds that are dispersed over different branches of the tree have been indicated by colored lines.
KAR
LES
DAL
IST
LAR
LIP
OVC
PIV
RUD
SKO
TSIR
BKR
KYM
DUB
PVO
LIK
RAC
SKZ
KCH
CHI
REC
ZUJ
SOR
BOV
JSO
TSIH
CRI
CIK
CRI(PAG)
IST
