# Supplementary material for: On the origin of European sheep as revealed by the diversity of the Balkan breeds and by optimizing population-genetic analysis tools
Source: Genet Sel Evol. 2020 May 14;52:25. doi: 10.1186/s12711-020-00545-7 (PMC7227234; doi:10.1186/s12711-020-00545-7)
Supplement: Supplementary file 7 — Additional file 7: Figure S3. FineStructure clustering of eastern and southeastern European sheep breeds. The color of each bin in the matrix indicates the number of “genomic chunks” copied from a donor (columns) to a recipient individual (rows). [file 12711_2020_545_MOESM7_ESM.pptx]

## Slide 1
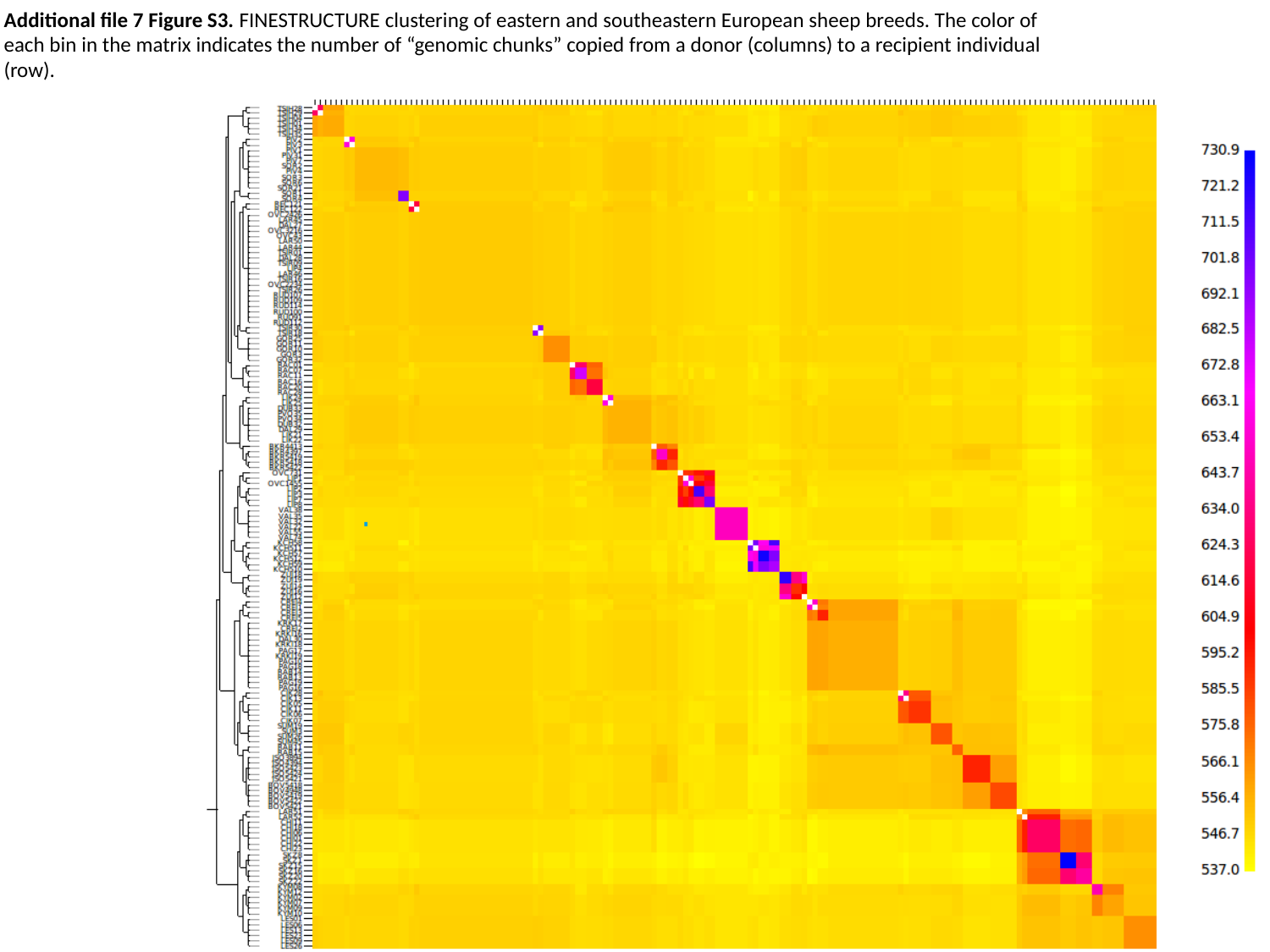

Additional file 7 Figure S3. FineStructure clustering of eastern and southeastern European sheep breeds. The color of each bin in the matrix indicates the number of “genomic chunks” copied from a donor (columns) to a recipient individual (row).
