# Supplementary material for: On the origin of European sheep as revealed by the diversity of the Balkan breeds and by optimizing population-genetic analysis tools
Source: Genet Sel Evol. 2020 May 14;52:25. doi: 10.1186/s12711-020-00545-7 (PMC7227234; doi:10.1186/s12711-020-00545-7)
Supplement: Supplementary file 8 — Additional file 8: Figure S4. Left panels: normal PCA plots of 525 sheep (≤ 6 per breed) including the inbred EFB, KCH, VBS. Right panels: supervised PCA of 546 sheep, including three mouflon populations, in which EFB, KCH and VBS as well as the mouflons have been excluded for calculation of the principal components (svPC1, svPC2 and svPC3). [file 12711_2020_545_MOESM8_ESM.docx]

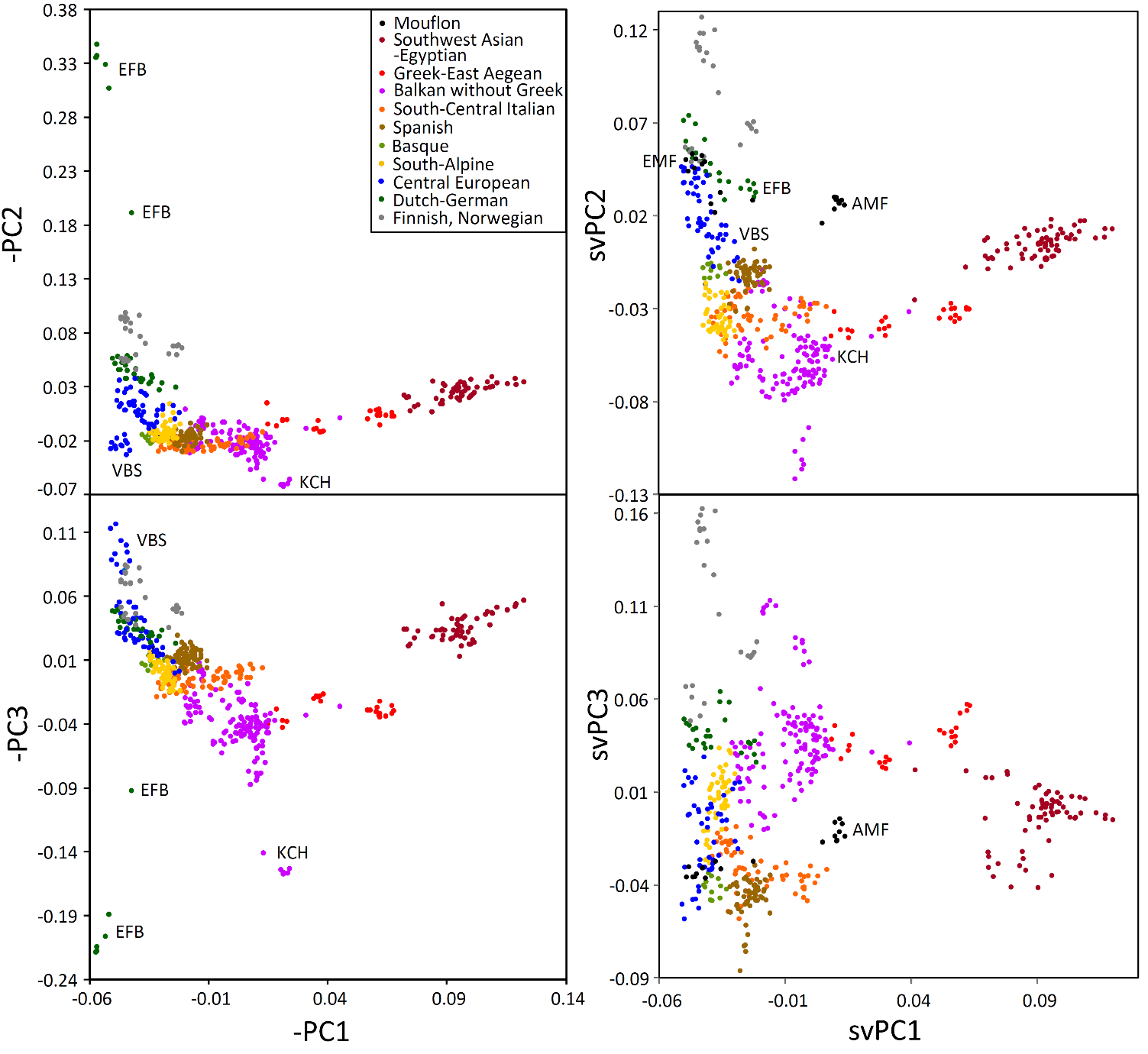


**Additional file 8 Figure S4.** Left panels: normal PCA plots of 525 sheep (≤6 per breed) including the inbred EFB, KCH, VBS. Right panels: supervised PCA of 546 sheep, including three mouflon populations, in which EFB, KCH and VBS as well as the mouflons have been excluded for calculation of the principal components (svPC1, svPC2 and svPC3).
