# Supplementary material for: On the origin of European sheep as revealed by the diversity of the Balkan breeds and by optimizing population-genetic analysis tools
Source: Genet Sel Evol. 2020 May 14;52:25. doi: 10.1186/s12711-020-00545-7 (PMC7227234; doi:10.1186/s12711-020-00545-7)
Supplement: Supplementary file 9 — Additional file 9: Figure S5. Supervised PCA of 546 animals as in Fig. 2b, showing svPC1 vs. svPC3 averaged per breed. [file 12711_2020_545_MOESM9_ESM.docx]

**Additional file 9 Figure S5.** Supervised PCA of 546 animals as in Fig. 2b, showing svPC1 vs svPC3 averaged for each breed.
