# Supplementary material for: On the origin of European sheep as revealed by the diversity of the Balkan breeds and by optimizing population-genetic analysis tools
Source: Genet Sel Evol. 2020 May 14;52:25. doi: 10.1186/s12711-020-00545-7 (PMC7227234; doi:10.1186/s12711-020-00545-7)
Supplement: Supplementary file 10 — Additional file 10: Figure S6. Spatial PCA of 507 domestic sheep without EFB, KCH and VBS, which were found to dominate the sPC2 and sPC3 just as for in the normal PCA (Additional file 8: Figure S4 left panels). The three methods of triangulation, indicated above the plots, give essentially the same results, which are similar to the supervised PCA pattern (see Additional file 8: Figure S4 right panels). [file 12711_2020_545_MOESM10_ESM.docx]

**Additional file 10 Figure S6.** Spatial PCA of 507 domestic sheep without EFB, KCH and VBS, which were found to dominate the sPC2 and sPC3 just as for in the normal PCA [see Additional file 8 Figure S4 left panels]. The three methods of triangulation, indicated above the plots, give essentially the same results, which are similar to the supervised PCA pattern in [Additional file 6 Figure S2 right panels].
