# Supplementary material for: On the origin of European sheep as revealed by the diversity of the Balkan breeds and by optimizing population-genetic analysis tools
Source: Genet Sel Evol. 2020 May 14;52:25. doi: 10.1186/s12711-020-00545-7 (PMC7227234; doi:10.1186/s12711-020-00545-7)
Supplement: Supplementary file 11 — Additional file 11: Figure S7. Supervised PCA of 546 animals in which the PC (svPC1, svPC2) were calculated based on the indicated fat-tailed, Nordic and Spanish sheep. [file 12711_2020_545_MOESM11_ESM.docx]

**Additional file 11 Figure S7.** Supervised PCA of 546 animals in which the PCs (svPC1, svPC2) were calculated based on the indicated fat-tailed, Nordic and Spanish sheeps.
