# Supplementary material for: On the origin of European sheep as revealed by the diversity of the Balkan breeds and by optimizing population-genetic analysis tools
Source: Genet Sel Evol. 2020 May 14;52:25. doi: 10.1186/s12711-020-00545-7 (PMC7227234; doi:10.1186/s12711-020-00545-7)
Supplement: Supplementary file 13 — Additional file 13: Figure S8. Neighbor-net graph of Reynolds’ distances between breeds or regional combinations of closely related breeds (see Additional file 12: Table S5). [file 12711_2020_545_MOESM13_ESM.docx]

**Additional file 13 Figure S8.** NeighborNet Graph of Reynolds’ distances between breeds or regional combinations of closely related breeds [see Additional file 12 Table S5A].
