# Supplementary material for: On the origin of European sheep as revealed by the diversity of the Balkan breeds and by optimizing population-genetic analysis tools
Source: Genet Sel Evol. 2020 May 14;52:25. doi: 10.1186/s12711-020-00545-7 (PMC7227234; doi:10.1186/s12711-020-00545-7)
Supplement: Supplementary file 14 — Additional file 14: Figure S9. Neighbor-net graphs of 17 regional groups of breeds (Additional file 12 B) with (A) AMF, (B) EMF, (C, D) both AMF and EMF; (D, E) pattern obtained by increasing the AMF-EFM distance in order to suppress the EMF-AMF clustering and to show different affinities of EMF and AMF for European domestic sheep. [file 12711_2020_545_MOESM14_ESM.docx]

(a) (b) (c) (d)

(e)

**Additional file 14 Figure S9.** NeighborNetwork graphs of 17 regional groups of breeds [see Additional file 12 Table S5B] with (a) AMF, (b) EMF, (c, d) both AMF and EMF; (d, e) pattern obtained by increasing the AMF-EFM distance in order to suppress the EMF-AMF clustering and to show different affinities of EMF and AMF for European domestic sheep.
