# Supplementary material for: On the origin of European sheep as revealed by the diversity of the Balkan breeds and by optimizing population-genetic analysis tools
Source: Genet Sel Evol. 2020 May 14;52:25. doi: 10.1186/s12711-020-00545-7 (PMC7227234; doi:10.1186/s12711-020-00545-7)
Supplement: Supplementary file 15 — Additional file 15: Figure S10. TreeMix trees without and with 6, 10 and 20 migrations and plots of the proportions of the variance explained (f-indices) and likelihoods at different m values. Coloured lines indicate inferred migrations with a weight according to the color scale. [file 12711_2020_545_MOESM15_ESM.docx]

***m*=0**

***m*=6**

***m*=10**

***m*=20**

**Additional file 15, Fig. S10.** TreeMix trees without and with 6, 10 and 20 migrations migrations and migrations and plots of the proportions of the variance explained (f-indices) and likelihoods at different k values. Colored lines indicate inferred migrations with a weight according to the color scale.

The tree generated without assuming migrations (*m* = 0) agrees with the NeighborNet graph (Additional file 14, Figure S9) and joins the feral EMF and SMF mouflons to the Nordic and Dutch-German Heath breeds as in Additional File 14 D and E. In the *m*=6 and *m*=10 graphs EMF and SMF are joined instead to Swiss breeds and in *m*=20 tree to the Basque sheep, which both have in the BSAA analysis about the same EMF ancestry as the Nordic and Heath breeds (Fig. 4). The strongest inferred gene flow is from European and Sardinian to Asian mouflons, reflecting the affinity of the wild Asian mouflon and their European feral descendants as indicated by a NeighborNet graphs ([25], Additional file 14 C). Another consistent gene flow between Sardinian white (SAW) and SMF is in agreement with the domestic introgression in the Sardinian mouflon population (Fig. 4, [46]), although at M=20 a gene flow is inferred from SMF to SAW instead of vice versa. Several inferred gene flows (between the EMF or EMF-SMF branch or EMF and Spael (NSP and NSW, *m* = 6, 10, 20), between EMF-SMF and Italian-Spanish breeds (M=10, 20) and between EMF-SMF and NWI) probably correspond to the EMF influence in European breeds as indicated by BSAA and the *f*4n across-breed profiles (Fig. 5).

The Merino introgressions indicated by BSAA and the *f*4n scan are in the TreeMix graphs represented by a gene flow from Merino to Swiss Alpine and Kamieniec (SWA and KAM). Several other inferred gene flows are between neighboring breeds.

The proportion of the variance explained by the plots increases form 0.90 at m=1 to 0.945 at m=12.
