# Supplementary material for: On the origin of European sheep as revealed by the diversity of the Balkan breeds and by optimizing population-genetic analysis tools
Source: Genet Sel Evol. 2020 May 14;52:25. doi: 10.1186/s12711-020-00545-7 (PMC7227234; doi:10.1186/s12711-020-00545-7)
Supplement: Supplementary file 16 — Additional file 16. Methodological comparisons and considerations [22, 50, 53, 54, 56, 64, 65, 77]. [file 12711_2020_545_MOESM16_ESM.docx]

**Additional File 16: Methodological considerations and comparisons.**

**1. ASD distances as quality control and checking breed differentiation**

Neighbor joining trees of ASD distances are not at all novel, but we recommend that their routine use for a rapid check of the dataset prior to further analysis. With several published and unpublished bead-array SNP datasets, we invariably found duplicate samples, outliers by either cross-breeding or mislabelling, unexpected subdivision of breeds and/or lack of differentiation at the breed level, which may confound downstream analyses. Most commonly, monophyletic clusters correspond to breeds and justify a simplification of PCA plots by averaging coordinates. This introduces clustering information in PCA plots as a convenient alternative to Discriminant Analysis of Principal Components [77]. In our study, we combined several Balkan breeds and other closely related breeds in order to simplify the phylogenetic analysis without losing any essential information. More sophisticated clustering may be based on sequential K-means and model selection analysis [77] or haplotype sharing of high-density SNP datasets [53].

**2. Coordination analysis and the detection of spatial patterns.**

Coordination analysis as PCA or MDS provides a survey of the data. At the same time, the main coordinates may reveal genetic clines that correspond to migration events. In the PCA including the Asian and European mouflons (Fig. 2a), the domestic breeds are lined-up according to a southeast to northwest cline between the fat-tailed and European mouflons. However, in the plot that contains only domestic sheep [see Additional file 8 Figure S4A], the components may be dominated by a few low-diversity breeds. This is observed in several published PCA plots and applies in our case to the influence of EFB, KCH and VBS. This can be circumvented by a supervised PCA (svPCA) in which these breeds are not used for calculating the supervised components. This returns a more even distribution of the breeds across the plots [see Additional file 8 Figure S4, left panels] and Fig. 2b with the low-diversity breeds plotted near other breeds from the same region and with the same east-to-west and south-to-north clines as described previously [22].

In Fig. 2c we calculated the svPC using only the genotypes of breeds with the northern, eastern and western most extreme locations, which emphasizes SNPs with the strongest drift of the allele frequencies during the migrations to these regions. We found that the interpolation of other breeds conforms to their geographical origin, which corresponds to plausible evidence for continuous clines as the result of the east-west and south-north gene flows, respectively. A similar pattern for the European breeds with the intersection of the two clines in northern Italy was obtained by using the Balkan Pramenka breeds as the eastern extreme (not shown).

It is noteworthy that these plots do not show a gene flow from Greece and other southern Balkan countries to the Italian and Iberian peninsula, although breeds from these regions are close to each other in the normal PCA plot. A possible explanation is that the migration across the Mediterrean coasts involved consecutive but separate events that cannot be captured in a single PCA coordinate. In general, we propose the identification of SNPs or haplotypes involved in specific clines as an approach to dissect a complex gene flow pattern.

For our dataset, supervised PCA combines geographic and genetic information more effectively than the spatial PCA (sPCA, [54]). In addition, it does not depend on any assumptions on spatial distances between breeds and the relative importance of terrestrial and maritime migration routes. Furthermore, it uses the original allele frequencies for the coordination analysis instead of combining genetic and spatial distances and thus maintains the direct relation with the genetic drift.

**3. Genetic distances between breeds**

Visualization of genetic distances between breeds gives additional information by showing phylogenetic relationships, even if this largely ignores reticulation events. We made pools of related breeds from the same region (Fig. 3) and [see Additional file 12 Tables S5, Additional file 13 Figure S8 and Additional file 14 Figure S9], which may be considered as proxies of the regional ancestor populations. This allows to reduce the complexity of the dataset and yields additional phylogenetic information by resolving a star-like network pattern (Fig. 3).

**4. Detection of ancestry and introgression**

Model-based clustering by Structure [56] or Admixture [50] highlights clusters of related individuals and may identify admixed individuals. However, because of the rapid development of livestock populations the real ancestors populations may very well be extinct. In addition, the inferred clusters are often biased towards homogeneous breeds with reduced diversity (e.g. the EFB signal in Fig. 4, 3^rd^ bar plot), which are unlikely to be ancestral. Conversely, individuals not belonging to an inferred cluster are shown to have an implausible mixed composition (as for AMF in the Fig. 4) . Even then, model-based clustering is a useful complement to coordination and phylogenetic analysis. However, in contrast to results with goat [64] or cattle [65] the AdmIxture patterns for sheep at k = 5 or higher (not shown) do not reproduce the regional clusters within Europe.

For breed-specific admixture analysis (BSAA), we carried out model-based clustering with subsets of SNPs that are specific for a given breed or population. This specificity is most likely generated by genetic drift in the source breed, but plausibly allows the detection of signatures of introgression into the target breeds. BSAA patterns with panels that differentiate between AMF and EMF admixture (Fig. 4) disclose an increasing influence of EMF from southeast to northwest Europe, which is consistent with the PCA (Fig. 2a) and the phylogenetic network (Fig. 3b). Remarkably, the EMF-specific SNP panel at k = 5 and the Merino-specific panel at k = 5 and k = 6 generate a better clustering according to geography than the Admixture plots with the genome-wide SNP panel.

We observed that the BSAA signals are consistent across those k values that show the required specificity and are also insensitive to the *F*_ST_ threshold that determines the number of informative SNPs. It is further encouraging that for the mouflons and for the Merino sheep, the BSAA is supported by an independent approach: a correlation of allele frequencies via normalized *f*4 (*f*4n) values (Fig. 5). BSAA seems to be more sensitive than the *f*4n scan across the breeds by giving higher estimates of the proportion of the genome that are contributed by introgression. Presumably, the admixture proportions are overestimated by BSAA because the non-admixed ancestors are not represented by a Structure-inferred cluster.

Furthermore, BSAA is better suited for the detection of the target rather than for the source of introgression. A BSAA specific for SWA or CIK, which both carry Merino introgression, generates weak signals for the donor breeds MER and MEE but also for other Spanish breeds (not shown). This reduced specificity reflects that introgression involves in addition to Merino-specific influence also Spanish influence. This has been excluded in the Merino panel, generated by contrasting Merino and Spanish non-Merino sheep, but not in the SWA- or CIK-specific panels selected by contrasting these breeds to nearby breeds without Merino introgression.

Admixture events generated by TreeMix are largely consistent with our other results, but admixture events involving several breeds are reproduced incompletely. Thus, the Merino introgression in several breeds corresponds to a Merino to SWA+KAM signal in the TreeMix analysis [see Additional file 15 Figure S10]. Furthermore, several directions of gene flow (for instance, from SWA to SMF and from NWI to EMF at *m*=20) are implausible.
